# The Complete Mitochondrial Genome of the Geophilomorph Centipede *Strigamia maritima*

**DOI:** 10.1371/journal.pone.0121369

**Published:** 2015-03-20

**Authors:** Helen E. Robertson, François Lapraz, Adelaide C. Rhodes, Maximilian J. Telford

**Affiliations:** 1 Department of Genetics, Evolution and Environment, University College London, Darwin Building, Gower Street, London, United Kingdom; 2 Center for Genome Research and Biocomputing, 2750 SW Campus Way, Oregon State University, Corvallis, Oregon, United States of America; Sichuan University, CHINA

## Abstract

*Strigamia maritima* (Myriapoda; Chilopoda) is a species from the soil-living order of geophilomorph centipedes. The Geophilomorpha is the most speciose order of centipedes with over a 1000 species described. They are notable for their large number of appendage bearing segments and are being used as a laboratory model to study the embryological process of segmentation within the myriapods. Using a scaffold derived from the recently published genome of *Strigamia maritima* that contained multiple mitochondrial protein-coding genes, here we report the complete mitochondrial genome of *Strigamia*, the first from any geophilomorph centipede. The mitochondrial genome of *S*. *maritima* is a circular molecule of 14,938 base pairs, within which we could identify the typical mitochondrial genome complement of 13 protein-coding genes and 2 ribosomal RNA genes. Sequences resembling 16 of the 22 transfer RNA genes typical of metazoan mitochondrial genomes could be identified, many of which have clear deviations from the standard ‘cloverleaf’ secondary structures of tRNA. Phylogenetic trees derived from the concatenated alignment of protein-coding genes of *S*. *maritima* and >50 other metazoans were unable to resolve the Myriapoda as monophyletic, but did support a monophyletic group of chilopods: *Strigamia* was resolved as the sister group of the scolopendromorph *Scolopocryptos sp*. and these two (Geophilomorpha and Scolopendromorpha), along with the Lithobiomorpha, formed a monophyletic group the Pleurostigmomorpha. Gene order within the *S*. *maritima* mitochondrial genome is unique compared to any other arthropod or metazoan mitochondrial genome to which it has been compared. The highly unusual organisation of the mitochondrial genome of *Strigamia maritima* is in striking contrast with the conservatively evolving nuclear genome: sampling of more members of this order of centipedes will be required to see whether this unusual organization is typical of the Geophilomorpha or results from a more recent reorganisation in the lineage leading to *Strigamia*.

## Introduction

*Strigamia maritima* is a geophilomorph centipede found widely along the coasts of North West Europe. It typically inhabits shingle beaches and stone crevices around the high tide line, where it feeds on crustaceans and insect larvae [1]. Geophilomorph centipedes demonstrate a number of unique features that make them a group of particular interest for evolutionary and developmental studies [2–4]. Unlike the vast majority of arthropod species, Geophilomorph members within the clade Adesmata, to which *S*. *maritima* belongs, show variability in adult segment number within the same species and between sexes [2]. Consequently, they represent an interesting group for studying developmental biology and the evolution of segmentation [2, 5]. Within the geophilomorphs, *S*. *maritima* is being used as a model species for investigating the evolution of segmentation within the arthropods [2] and understanding developmental processes within the myriapods [3]. A number of studies have been carried out to characterise its embryological development [3, 6–8], and in particular the process of trunk segmentation [5, 9–15].

*S*. *maritima* is the first centipede, and indeed the first myriapod, with a completely sequenced nuclear genome [16]. As part of the genome sequencing effort, one of the assembled scaffolds was discovered to contain numerous mitochondrial protein-coding genes, and it was deemed likely that this scaffold represented the assembled mitochondrial genome. We have used this assembled contig sequence from *Strigamia* as the framework for resequencing the complete mitochondrial genome of this animal. We use this complete sequence and gene order to evaluate whether this mitochondrial genome is useful as a phylogenetic marker for testing ideas about the phylogenetic position of the geophilomorphs within the centipedes, and the centipedes within the wider context of the myriapods and arthropods.

### The position of the myriapods within the euarthropods

The relationships between the four euarthropod classes—Chelicerata (arachnids, pycogonids and horse shoe crabs); Crustacea (crabs, copepods etc); Myriapoda (e.g. centipedes and millipedes) and Hexapoda (including insects)—have long been a controversial topic within evolutionary biology. Mitochondrial gene arrangements and molecular phylogenies have convincingly shown that the crustaceans and hexapods form a monophyletic group, the Pancrustacea, in which the hexapods constitute a branch within a larger ‘pancrustacean’ clade [17]. This well-supported pancrustacean alliance breaks up the old Atelocerata/Uniramia group of Hexapoda and Myriapoda, and the most contentious remaining issue concerns the position of the Myriapoda relative to Pancrustacea and Chelicerata [18]. The traditional grouping of myriapods, hexapods and crustaceans into a group termed the Mandibulata is most obviously based on their shared morphological feature of the post-tritocerebral appendage forming the mandible; chelicerates lack a mandible, and the homologous segment has a pair of walking legs [18]. In contrast, phylogenies compiled from a range of molecular data have tended instead to unite myriapods with the chelicerates in the Myriochelata, rather than to the other mandibulates. [18–20]. Incongruence of morphology and some molecular data have prompted a number of careful studies of the data leading to the suggestion that the support for a Myriochelata grouping may have arisen as a result of systematic error [18]. Resolving this through careful outgroup selection [19] and removing genes with a high rate of nonsynonymous change [21] demonstrated that the strongest phylogenetic signals were in fact in support of Mandibulata. This evidence, and additional analyses of molecular data, indicates a degree of support for Myriapoda as the sister group to Pancrustacea, within a monophyletic Mandibulata [19, 22]. Despite this, the position of the Myriapoda within the Arthropoda remains difficult to resolve.

### The position of the chilopods within the myriapods

Extant myriapods are represented by two main groups: the herbivorous millipedes (Diplopoda), and the carnivorous centipedes (Chilopoda). In addition there are two minor groupings: Symphyla and Pauropoda. Whilst the monophyly of each of the four myriapod groups is well-supported by both molecular and morphological studies [23], the inter-relationships of the myriapod classes remain difficult to resolve [24]. Morphological and developmental evidence has traditionally placed the Pauropoda and Diplopoda together as sister lineages in the Dignatha; Symphyla and Dignatha together have been classified as the Progoneata, named for the common presence of an anterior gonopore, with Chilopoda as sister group. In contrast to this, molecular analyses have instead indicated a sister clade relationship between Symphyla and Pauropoda, together forming the Edafopoda [25–27]. Both morphological [28] and molecular [24] studies have yielded a degree of support for a paraphyletic Myriapoda, placing the Chilopoda as sister group to the Chelicerata, and Diplopoda as sister group to Chilopoda + Chelicerata. However, a number of molecular analyses demonstrate strong evidence for the monophyly of the myriapods [25, 26, 29]. More recent phylogenomic analyses support a monophyletic Myriapoda, but place the symphylans as sister group to the three other myriapod classes [30, 31].

### The position of the geophilomorphs within the chilopods

The Chilopoda comprises approximately 3000 species within five extant orders: Scutigeromorpha, Lithobiomorpha, Craterostigmomorpha, Scolopendromorpha, and the most diverse order, the Geophilomorpha, to which *Strigamia maritima* belongs [32]. Relationships between chilopod clades seem well resolved from morphological characters and molecular data derived predominantly from single nuclear DNA markers [33]. Molecular data sets support the basal split of the Chilopoda into two evolutionary lineages: the Notostigmophora (= Scutigeromorpha) and Pleurostigmomorpha (the remaining four orders including geophilomorphs), and do not support the alternative hypothesis that Geophilomorpha are the sister group to all other chilopod orders [32–34].

In this study, we describe the complete mitochondrial genome of the centipede *Strigamia maritima*. No geophilomorph mitochondrial genome has been published to date. Here we analyse the gene content and gene order of the *S*. *maritima* mitochondrial genome in comparison to other arthropod species, and describe the results of a phylogenetic analysis using sequence alignments from mitochondrial protein-coding genes.

## Materials and Methods

### Initial Sequence from genome scaffold

Within the *S*. *maritima* whole genome sequence, the scaffold scf718000124766, 23.9kb in length, was found by BLAST to contain a series of mitochondrial protein-coding genes. Closer examination showed atypical large non-coding regions at each end of the scaffold and multiple frameshift errors within protein-coding genes, probably the results of assembly errors within the scaffold. In order to correct possible errors both of assembly and of single mis-read nucleotides we designed PCR primers covering most of the length of the scaffold sequence, and in particular covering all areas containing apparent frameshifts.

### DNA Extraction, Primer Design and PCR

DNA was isolated from a population of *Strigamia maritima* living in the wild on the East coast of Scotland [5] and provided to us by the Akam lab. The DNA used came from a pooled sample of animals, and all sequencing was carried out directly on PCR fragments amplified from this pool. In cases where there is heterozygosity in the population, therefore, the sequence we report will show the most frequently occurring alleles in the PCR product which is likely to represent the highest frequency allele in the population used. Centipedes are not regulated in directive 2010/63/EU of the European Parliament or the UK Animals (Scientific Procedures) Act 1986, but care was taken to minimise potential suffering of the animals.

PCR primers were designed using Primer3 [35] based on the initial 23.9kb scaffold, with the objectives of: i) verifying total genome length, ii) linking the two ends of the mitochondrial genome to produce a closed circle, and iii) correcting sequencing errors within the scaffold sequence. Primer pair sequences are available in the supporting information (S1 Table). Outward facing PCR primers were first designed within conserved gene regions at either end of the scaffold to link both ends of the sequenced genome (to ‘close’ the circular genome). Within the resulting circular genome (corrected length 14,638 base pairs), PCR primers were designed to amplify the entire sequence in nine overlapping fragments of approximately 2kb each. Where possible, primers were located within conserved protein-coding gene sequences. Of these nine fragments, all but one were successfully amplified. Following gene annotation of the new DNA sequence, likely erroneous stop codons were identified remaining within the coding sequence of *nad6*. New primers were designed to amplify this region allowing us to correct these remaining errors.

All PCRs were performed using the GeneAmp PCR System 2700 (Applied Biosystems, California, USA). PCRs were carried out using the Expand Long-Range PCR Kit (Roche Life Sciences, Penzberg, Germany), following manufacture’s recommendations for a 50μl reaction set-up. The Expand Long-Range kit was used owing to its optimisation for amplification of long PCR products, and the high proofreading activity of the polymerase. Cycling was set up as follows: 92°C for 2 min (initial denaturation); 15 cycles of: 92°C for 10 sec (denaturation); 57°C for 15 sec (annealing), 68°C at initial elongation time (approximated as 1 min per 1000 nucleotides to be amplified); 2 cycles each of: 92°C for 10 sec (denaturation), 57°C for 15 sec (annealing), 68°C at 40 sec longer than the initial elongation time, repeated at elongation times increasing by 40 sec intervals for a total of 14 further cycles (two cycles each at seven increasing elongation times). A final elongation stage at 68°C for 7 min was followed by a 4°C ‘hold’ stage. Amplified products were size separated on ethidium-bromide stained TAE 1% agarose gel and visualised. Successfully amplified products were purified using the High Pure PCR Product Purification Kit (Roche Life Sciences, Penzberg, Germany) with the manufacturer-recommended protocol and sequenced using fluorescent sanger sequencing. Only amplifications which resulted in a single strong band on the agarose gel were purified and sequenced.

### Data Assembly and Gene Annotation

For all successful PCR amplifications, forward sequencing results, and the reverse complement of reverse sequencing results, were merged together using the EMBOSS 6.3.1 DNA merger program (http://bioinfo.nhri.org.tw/cgi-bin/emboss/merger), to produce the whole sequenced fragment. The sequence of the amplified closed genome fragment replaced the sequence originally found in the end 4kb and front 3kb regions to correct the length of the mitochondrial genome. The resulting 14,638 base pair circular genome was then used as a point of reference to align each of the sequencing results for the ~2kb fragments (I-VII and IX), resolving the final *Strigamia* genome as 14,983 base pairs in length. Our sequencing results for each of the genes covered by these fragments were compared to the initial sequence to correct any remaining frameshifts, with subsequent results from the *NADH dehydrogenase subunit 6* (*nad6*) fragment fixing the remaining frameshift mutations within this gene. A new consensus sequence for each gene, based on these results, was generated. In the case of nucleotide ambiguity between the original assembled sequence and new sequencing results, the new sequencing results took preference.

### Phylogenetic Inference

Phylogenetic analyses were carried out using a concatenated amino acid alignment of all thirteen protein-coding genes from the *S*. *maritima* mitochondrial genome. The *S*. *maritima* protein-coding sequences were first translated using the standard invertebrate mitochondrial genetic code, and the amino acid sequences for each gene were aligned to orthologs from other taxa using MUSCLE [36] (S2 Table). The resulting alignments were trimmed using trimA1 1.2rev59 (with standard settings) [37], and the alignments finally concatenated to produce an alignment of 3407 amino acids from 54 species.

Bayesian analyses was carried out using the site-heterogeneous CAT-GTR mixture model in the PhyloBayes 3.3f software package [38] to allow site-specific amino acid preferences. Four discrete gamma categories are used to distinguish between site-specific rate heterogeneity across the sequence. This model is implemented within a Monte Carlo Markov Chain (MCMC) algorithm, using PhyloBayes. For each alignment, two independent runs were performed for >14,000 cycles and the summary tree calculated with a ‘burn in’ of 3000 cycles.

Trees were also reconstructed using the maximum likelihood approach using PhyML v 3.0. [39]. The MTArt substitution model was selected, the proportion of invariable sites was estimated and a gamma distribution with 4 categories used. An approximate likelihood ratio test using SH-like supports was conducted to provide estimates of support for clades on the best tree.

## Results

### Organisation of the genome and genes

**Fig 1:**
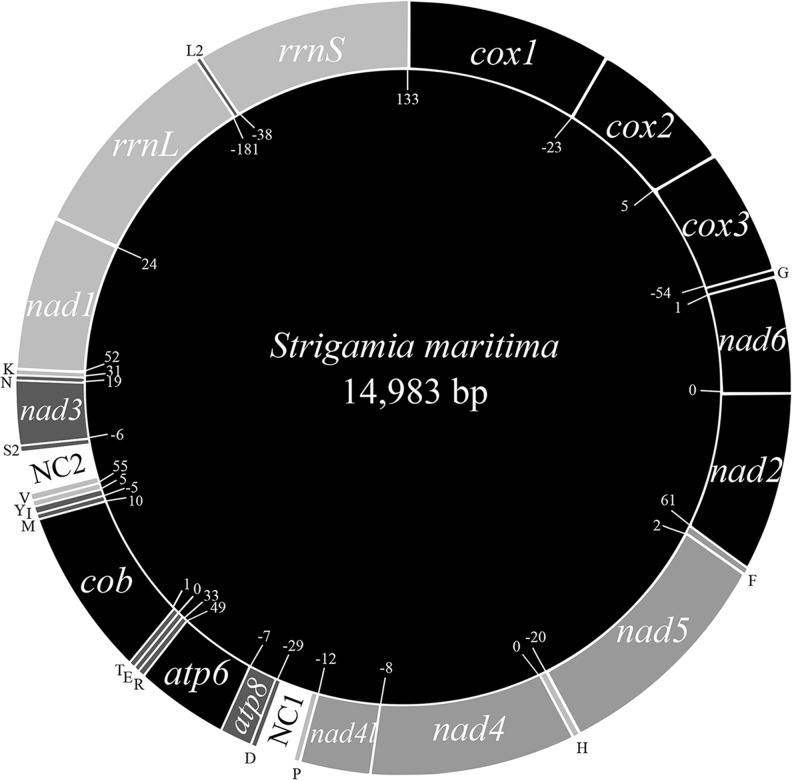
Overview of the mitochondrial genome of *Strigamia maritima* (Myriapoda: Chilopoda), genes not drawn to scale. Numbers inside the circle show intergenic spaces (positive values) or intergenic overlaps (negative values). Protein-coding genes are denoted by three letter abbreviations, ribosomal genes denoted by four letter abbreviations. tRNAs are indicated by single uppercase letters.

The circular, double stranded mitochondrial genome of *S*. *maritima* is 14,983 base pairs long: 8,925 base pairs shorter than the original contig from the genome assembly (Fig. 1, Table 1). The erroneous additional bases in the original scaffold showed similarity to TY1/Copia-like retrotransposons and our success in closing the circular molecule demonstrates that these derive from incorrect assembly. Our re-sequencing allowed us to correct 15 frame shifts and to correct 584 other incorrectly identified and/or missing nucleotides. The genome contains both the small and large subunit of ribosomal RNA (*rrnS* and *rrnL*), thirteen protein-coding genes (*cytochrome c oxidase* (*cox*) *1*, *2*, *3*; *apocytochrome b* (*cob*); *NADH dehydrogenase* (*nad*) 1, *2*, *3*, *4*, *4l*, 5, *6*; and *ATP synthase F0* (*atp*) 6 and 8) and two large non-coding regions. Sixteen tRNAs were identified using the MiTFi program within MITOS [40, 41]: *trnG*, *trnF*, *trnH*, *trnP*, *trnD*, *trnR*, *trnE*, *trnT*, *trnM*, *trnI*, *trnY*, *trnV*, *trnS2*, *trnN*, *trnK*, *trnL2*. Sequences resembling *trnW*, *trnQ* and *trnA* could only be predicted in the same sequence as *trnI*, *trnE* and *trnT*, respectively, and with low e-values. No credible sequence could be predicted for *trnC*, *trnL1* or *trnS1* using MiTFi or any alternative tRNA prediction software (ARWEN [42], tRNAscan-SE [43]). The predicted sequence for *trnG* is found entirely within the sequence for *cox3*, on the same strand; *trnH* and *trnP* also have partial overlap on the same strand with *nad5* and *nad4l*, respectively. Whilst the predicted sequence of *trnL2* overlaps largely with *rrnL*, they are found on opposite strands (Table 1).

**Table 1:** Organisation of the *Strigamia maritima* mitochondrial genome.

Gene	Strand	Start position	End position	Length	Start codon	Stop codon	Intergenic nucleotides
*cox1*	+	1	1557	1557	ATT	TAA	
*cox2*	+	1532	2215	684	ATG	TAG	-23
*cox3*	+	2221	3063	843	ATG	TAA	+5
*trnG*	+	3010	3058	49			-54
*nad6*	+	3060	3524	464	ATA	TAG	+1
*nad2*	+	3525	4487	963	ATT	TAA	0
*trnF*	-	4547	4603	57			+61
*nad5*	-	4606	6306	1701	ATG	TAG	+2
*trnH*	-	6287	6347	61			-20
*nad4*	-	6348	7664	1317	ATG	TAA	0
*nad4l*	-	7658	7921	264	ATT	TAA	-8
*trnP*	-	7909	7972	64			-13
*NC1*		7973	8414	442			0
*trnD*	+	8415	8489	75			0
*atp8*	+	8461	8622	162	ATA	TAA	-29
*atp6*	+	8616	9281	666	ATG	TAA	-7
*trnR*	+	9331	9375	45			+49
*trnE*	+	9409	9454	46			+33
*trnT*	+	9455	9506	52			0
*cob*	+	9508	10641	1134	ATC	TAG	+1
*trnM*	+	10652	10710	59			+10
*trnI*	+	10706	10759	54			-5
*trnY*	-	10765	10825	61			+5
*trnV*	-	10880	10937	58			+55
*NC2*		10938	11331	394			0
*trnS2*	+	11332	11387	56			0
*nad3*	+	11382	11732	351	ATT	TAA	-6
*trnN*	+	11752	11799	48			+19
*trnK*	-	11831	11888	58			+31
*nad1*	-	11942	12862	921	ATT	TAG	+52
*rrnL*	-	12888	14258	1371			+24
*trnL2*	+	14078	14148	71			-181
*rrnS*	-	14111	14850	740			-38

Intergenic nucleotides shown as gaps (positive values) or overlap (negative values) between consecutive genes.

**Fig 2:**
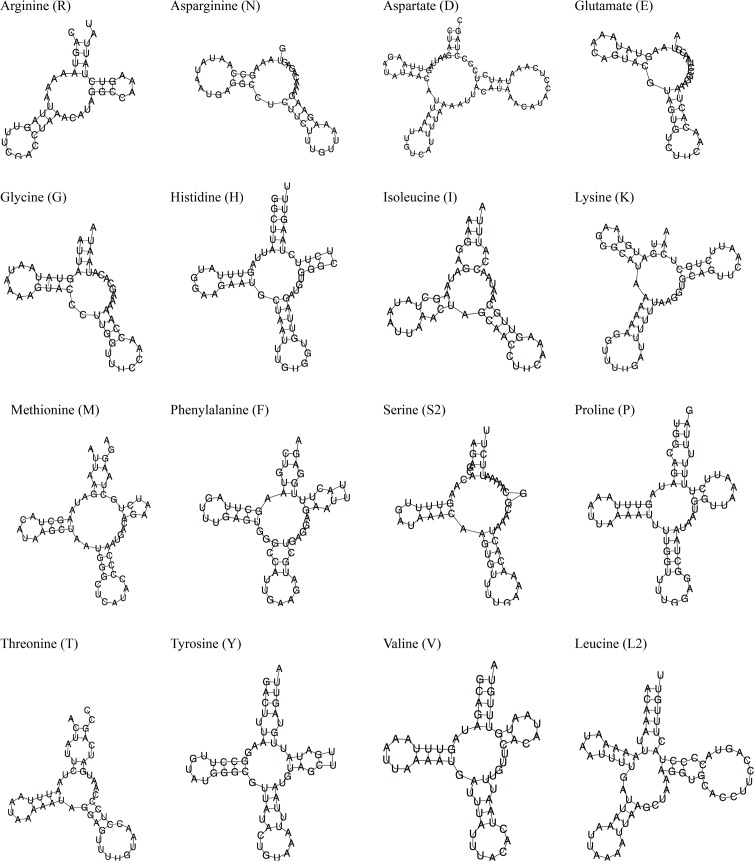
Putative secondary structures of tRNAs from the mitochondrial genome of *Strigamia maritima* as predicted by MiTFi [40, 41].

Secondary structures were determined for the sixteen tRNAs which could be reliably identified using MiTFi (Fig. 2). Clear deviations from the classical ‘cloverleaf’ tRNA secondary structure are observed in many of the *S*. *maritima* tRNA putative secondary structures. One or more of the four loops are commonly totally or partially missing. The DHU loop is entirely missing in *trnR*, and the TΨC loop entirely missing in the predicted structure for *trnN*, *trnE*, *trnG*, *trnM*, *trnS2* and *trnT*. The structure of the acceptor stem is severely truncated or entirely lacking in *trnR*, *trnN*, *trnD*, *trnE*, *trnG*, *trnI*, *trnK* and *trnT*. Within their predicted secondary structures, all tRNAs appear to have mismatched nucleotides and a combination of enlarged or shrunken loops and/or truncated stems.

Mitochondrial genes are transcribed from both strands, with the strand bearing the most protein-coding sequences designated the ‘plus’ strand. *cox1–3*, *nad6*, *nad2*, *atp8*, *atp6*, *nad3*, as well as *trnD*, *trnR*, *trnE*, *trnT*, *trnM*, *trnI*, *trnS2* and *trnN* are on the plus strand; the five remaining protein-coding genes, both ribosomal genes, and the remaining identified tRNAs, are on the minus strand. Coding DNA accounts for 92.1% of the genome. Of this, and taking into account overlapping regions, protein-coding genes account for 79.31% of the coding DNA, ribosomal genes for 15.24% and tRNA genes for 6.62%. Initiation codons in all *S*. *maritima* protein-coding genes are ATN: ATT (x5), ATG (x5), ATA (x2) and ATC (x1). Stop codons for all genes were complete: TAA (x8) and TAG (x5) (Tables 1 and 2). Total codon usage across all protein-coding genes is shown in Table 2.

**Table 2:** Total codon usage across the protein-coding genes of the *Strigamia* mitochondrial genome.

Codon	Aa	Frequency	Codon	Aa	Frequency	Codon	Aa	Frequency	Codon	Aa	Frequency
**TTT**	F	226	**TCT**	S	96	**TAT**	Y	82	**TGT**	C	25
**TTC**	F	61	**TCC**	S	31	**TAC**	Y	53	**TGC**	C	16
**TTA**	L	184	**TCA**	S	71	**TAA**	STOP	8	**TGA**	W	71
**TTG**	L	82	**TCG**	S	10	**TAG**	STOP	5	**TGG**	W	35
**CTT**	L	81	**CCT**	P	44	**CAT**	H	39	**CGT**	R	12
**CTC**	L	23	**CCC**	P	54	**CAC**	H	43	**CGC**	R	5
**CTA**	L	136	**CCA**	P	59	**CAA**	Q	61	**CGA**	R	35
**CTG**	L	19	**CCG**	P	6	**CAG**	Q	15	**CGG**	R	10
**ATT**	I	195	**ACT**	T	41	**AAT**	N	47	**AGT**	S	25
**ATC**	I	91	**ACC**	T	55	**AAC**	N	56	**AGC**	S	15
**ATA**	M	169	**ACA**	T	129	**AAA**	K	65	**AGA**	S	60
**ATG**	M	57	**ACG**	T	8	**AAG**	K	17	**AGG**	S	15
**GTT**	V	117	**GCT**	A	62	**GAT**	D	32	**GGT**	G	59
**GTC**	V	16	**GCC**	A	57	**GAC**	D	35	**GGC**	G	47
**GTA**	V	79	**GCA**	A	83	**GAA**	E	48	**GGA**	G	67
**GTG**	V	53	**GCG**	A	14	**GAG**	E	37	**GGG**	G	122

Standard one-letter abbreviations for amino acids (Aa) using invertebrate mitochondrial genetic code.

The A+T content of the total genome is 64.02%, which is lower than the percentage found in the mitochondrial genome of the centipedes *Lithobius forficatus* (67.9%) [44] and *Scutigera coleoptrata* (69.4%)[45], the pauropod *Pauropus longiramus* (72.9%) [27], the symphylan *Scutigerella causeyae* (72.6%)[20] and the millipede *Thyropygus* sp. (67.8%) [46]; close to that of the millipede *Narceus annularis* (63.7%) [46]; and higher than that of the millipede *Antrokoreana gracilipes* (62.1%) [47]. The A+T content of the coding portion of the genome is 63.5%. Average A+T content across the thirteen protein-coding genes is 62.8%; lower than the 68.58% average A+T content of the two ribosomal genes. As a result of the high A+T content of the mitochondrial genome, the most frequently occurring codons across the protein-coding genes are those comprised of A and T nucleotides: TTT (x 226), ATT (x 195), TTA (x 184) and ATA (x 169) (Table 2). Two main non-coding regions, NC1 and NC2 (Table 1) were found to have a higher A+T content than that of the total genome: 71.27% for NC1 and 71.07% for NC2. The compositional difference between the non-coding regions and the genome as a whole is statistically significant (NC1, χ2 = 9.503, p<0.01; NC2, χ2 = 7.991, p<0.01); consequently, these two regions are proposed as control regions [48]. Eight other short non-coding regions, ranging in length from 19 to 131 nucleotides are also found throughout the genome. None of these regions have an A+T content that is statistically significantly higher than that of the genome as a whole.

Nucleotide composition of the plus strand is as follows: A = 38.96%; T = 25.06%, C = 23.90% and G = 12.08%. Base compositional bias between the two strands can be measured as GC- and AT- skew, where GC-skew = (G—C)/(G + C) and AT-skew = (A—T)/(A + T)[49]. Using these formulae, skew values are generated ranging in value from-1 to +1; an absolute value closer to 1 indicates compositional asymmetry between the two stands, whilst a value of 0 indicates that distribution is equal between the strands. For the *S*. *maritima* plus strand, GC-skew = -0.33 and AT-skew = 0.22, showing asymmetry in nucleotide composition between the two strands. The absolute GC-skew value is higher than that found in *Thyropygus sp* (-0.29) [46], *L*. *forficatus* (-0.27) [44] and *S*. *coleoptrata* (-0.31) [45], but lower than that of *N*. *annularis* (-0.40) [46]. Absolute AT-skew is higher than that in *Thyropygus sp* (0.08) [46], *L*. *forficatus* (0.09) [44], *S*. *coleoptrata* (0.04) [45] *N*. *annularis* (0.07) [46] and *S*. *causeyae* (-0.12) [20].

### Gene order

**Fig 3:**
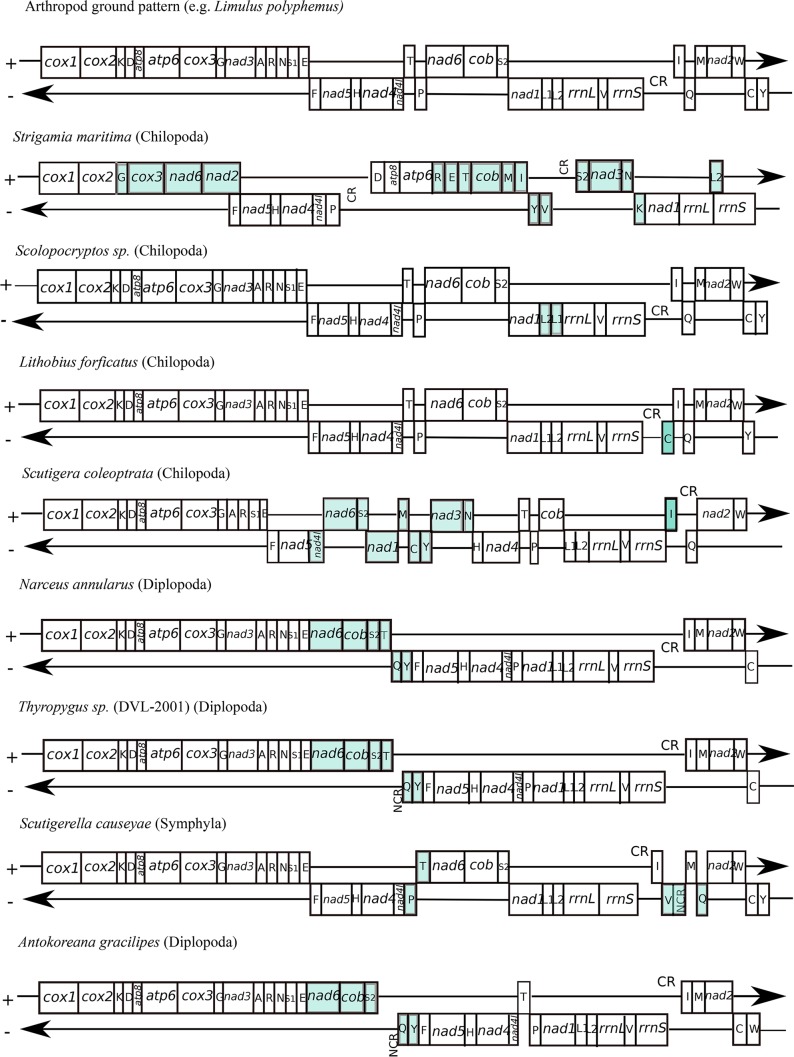
Comparisons of gene orders in the mitochondrial genomes of *Strigamia maritima*, the arthropod ‘ground-plan’ (*Limulus polyphemus*), three further chilopods, three diplopods and a symphylan. Genes are not drawn to scale; shaded genes indicate those that have moved from the original arthropod ‘ground-plan’.

The overall arrangement of genes around the *S*. *maritima* mitochondrial genome is unique compared to other arthropod species or to any other metazoan mitochondrial genome studied (Fig. 3). Genes of the same transcriptional polarity are clustered together, with the exception of *trnL2*, which overlaps with genes on the opposite strand. Four blocks of protein-coding genes follow the arthropod ‘ground plan’ (Fig. 3): *cox1*-*cox2* (plus strand); *trnF*-*nad5*-*trnH*-*nad4*-*nad4l*-*trnP* (minus strand); *trnD*-*atp8*-*atp6* (translocated towards the 3’ end of the plus strand); and *nad1*-*rrnL*-*rrnS* (translocated towards the 5’ end of the minus strand). The composition of the rest of the genome has a gene order which is completely unique to *Strigamia*: *nad6* and *nad2* have been rearranged adjacent to the *cox1*-*cox2*-*trnG*-*cox3* block at the 5’ end of the plus strand; *cob* has rearranged to the 3’ end of the *trnD*-*atp8*-*atp6* block, with the addition of *trnR*, *trnE*, *trnT* on the 5’ side, and *trnM* and *trnI* on the 3’ end; and *trnS2-nad3-trnN* is a novel arrangement on the minus strand. Two main non-coding control regions are proposed, one between *trnP* and *trnD*, and the other between *trnV* and *trnS*2. The location of these is unique to *Strigamia*. Compared to other arthropod species, the genes of *S*. *maritima* have a large degree of overlap and of intergenic space (Table 1).

### Phylogenetic Analysis

**Fig 4:**
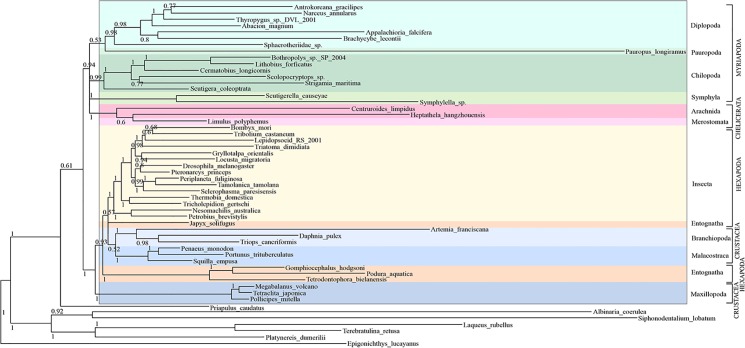
Bayesian phylogenetic analysis of mitochondrial protein-coding genes from Arthropoda species including *S*. *maritima*. Support values at nodes are Bayesian Posterior Probability (BPP). Myriapoda and Chelicerata, ‘Myriochelata’ (BPP = 0.94) resolved as the sister group to Pancrustacea (Crustacea and Hexapoda, BPP = 1.0). A monophyletic Chilopoda is resolved with BPP = 0.99, within which Scutigeromorpha (*Scutigera coleoptrata*) are resolved as the sister group to the three remaining chilopod orders represented in our phylogeny (Lithobiomorpha, (*Bothropolys sp*., *Lithobius forficatus* and *Cermatobius longicornis*); Scolopendromorpha (*Scolopocryptos sp*.) and Geophilomorpha (*Strigamia maritima*)) with BPP = 1.

**Fig 5:**
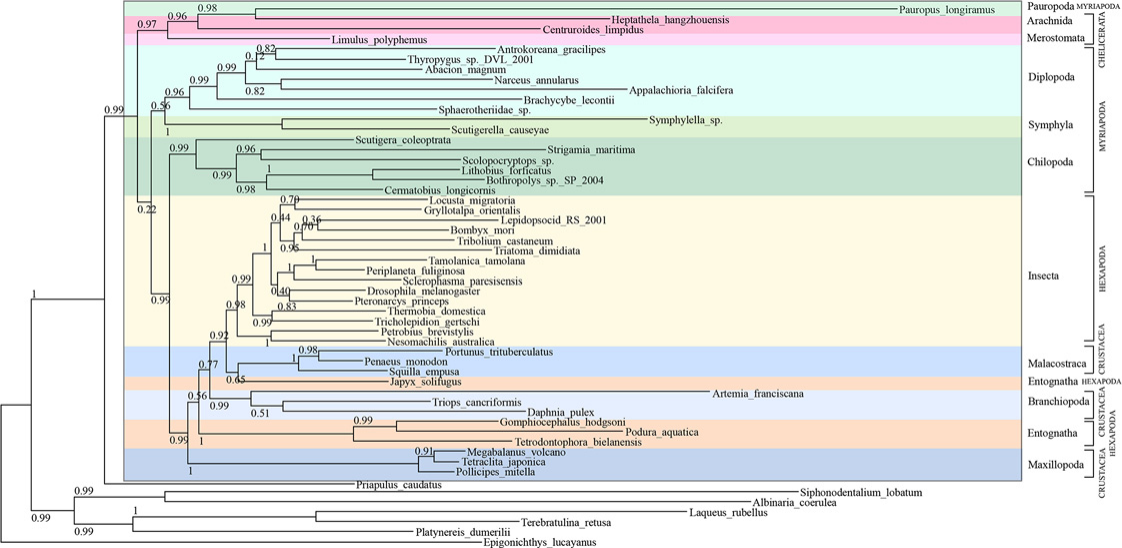
Maximum Likelihood phylogenetic analysis of mitochondrial protein-coding genes from Arthropoda species including *S*. *maritima*. Support at nodes are SH-like support values. A monophyletic Chilopoda is resolved as sister group to Pancrustacea (SH-like support = 0.99) and Pauropoda (represented by *Pauropus longiramus*) placed as sister group to Chelicerata (SH-like support = 0.97). Within the Chilopoda, Scutigeromorpha (*Scutigera coleoptrata*) are resolved as sister group to the three other chilopod orders represented in our phylogeny (Lithobiomorpha, (*Bothropolys sp*., *Lithobius forficatus* and *Cermatobius longicornis*); Scolopendromorpha (*Scolopocryptos sp*.) and Geophilomorpha (*Strigamia maritima*) with SH-like support = 0.99.

Using a data set of 54 species, PhyloBayes Bayesian and Maximum Likelihood (ML) phylogenetic analysis was performed using conserved blocks of amino acid alignments of protein-coding genes (Fig. 4). The arthropod portion of the tree is rooted with the deuterostome cephalochordate *Epigonichthys lucayanus* as well as five lophotrochozoans (two molluscs, one annelid and two brachiopods) and the ecdysozoan priapulid *Priapulus caudatus*. In the Bayesian phylogeny, (Fig. 4) Myriapoda and Chelicerata, ‘Myriochelata’, are resolved as the sister group to Pancrustacea, with Bayesian Posterior Probabilities (BPP) of 0.94 (Myriochelata) and BPP = 1 (Pancrustacea). Within the clade of Myriochelata the chelicerates are supported as monophyletic with maximum support, but the analysis did not resolve the myriapods as monophyletic. Three myriapod clades are resolved, however: Chilopoda (BPP = 0.99); Diplopoda plus Pauropoda (BPP = 0.53; Diplopoda alone BPP = 0.98) and Symphyla (BPP = 1). Our ML analysis (Fig. 5) resolves a monophyletic Mandibulata (apart from the anomalous pauropod) but shows a paraphyletic Myriapoda, placing the Chilopoda as sister group to Crustacea + Hexapoda with SH-like support value 0.99, and the Pauropoda (represented by *Pauropus longiramus*) as sister group to the Chelicerata (SH-like support = 0.97). The internal relationships of the four Chilopoda orders in both of our phylogenetic analyses corroborates the consensus opinion on centipede relationships derived from other molecular analyses [33].

## Discussion

### Genome composition and tRNAs

All thirteen protein-coding genes and both ribosomal genes were found in the *S*. *maritima* mitochondrial genome. Only sixteen of the standard 22 tRNAs were identified. The lack of detectable tRNAs using our bionformatic analysis may be due to a truncation of tRNA sequences and/or asymmetry of tRNA secondary structure such as have been previously found in other arthropod genomes [20, 27]. *trnW*, *trnQ* and *trnA* could only be predicted within the same sequence as *trnI*, *trnE* and *trnT*, respectively, which demonstrates a degree of ambiguity in determining tRNA sequences for *Strigamia* using bioinformatic approaches.

For the sixteen tRNA sequences that could be identified, it is clear that they do not all conform to the canonical cloverleaf-shaped secondary structure of tRNAs: for all tRNAs, stems contain mismatches and/or are truncated, and one or more loop may be either missing or greatly modified (Fig. 3). This situation is not unique to *Strigamia*: complete loss of the TΨC loop in tRNA has been described in a number of metazoan mitochondrial genomes, including the jumping spider *Habronattus oregonensis* (Chelicerata) [50]. As in *Strigamia*, *H*. *oregonensis* has asymmetric tRNA sequences which cannot be folded into a typical cloverleaf-secondary structure [50]. In addition, many of its tRNAs also lack a fully paired acceptor stem. In both *Strigamia* and *Habronattus*, tRNAs overlap with other RNAs or protein-coding genes on the same or the opposite strand, which could result in a truncation of what would normally be the acceptor stem of the tRNA. It is also possible that the 3’ portion of the acceptor stem may be formed post-transcriptionally, as in the centipede *Lithobius forficatus* [44]. Truncated tRNAs as a result of overlapping genes may be the result of a tendency to reduce mitochondrial genome size, as has also been proposed for the myriapod *Pauropus longiramus* [27], but the evolutionary advantage of this is uncertain. One proposed outcome of incomplete tRNAs is that they cause an accumulation of deleterious mutations at a faster-than-normal rate, leading to a potential ‘mutational meltdown’[51]. Theoretically, if posttranscriptional modification could keep up with the accumulation of mutations, as well as reduce the mitochondrial genome size, the truncated tRNAs would be retained whilst reducing the mitochondrial genome size as observed.

### Gene Order

Previous comparisons across the arthropods, and more widely within the Ecdysozoa, proposed that the ancestral arthropod mitochondrial genome has a gene arrangement identical to that found in *Limulus polyphemus* [52]. Mitochondrial gene order can be an informative phylogenetic tool, and a significant finding of this study is that gene order in *S*. *maritima* is notably different from that of any other myriapod, or indeed any other metazoan species, to which it can be compared. Whilst small regions of gene order in *Strigamia* follow that of the arthropod ‘ground pattern’, (for example, *trnF-nad5-trnH-nad4-nad4L* on the minus strand), other sections are completely rearranged without a precedent among metazoans.

Gene order rearrangement is most commonly thought to occur via a ‘duplication and deletion’ model. This proposes that the random duplication of part of the mitochondrial genome occurs as a result of slipped-strand mispairing or an error during replication termination. Following this, one of the gene copies is deleted. If it is the original copy that becomes deleted this results in a change in gene order [53]. Evidence for this model is provided by mitochondrial genomes with duplicated regions including at least one protein-coding or rRNA gene [53, 54].

In a recent study concerning the house centipede *Scutigera coleoptrata* (Scutigeromorpha), a novel mitochondrial gene arrangement could only be explained by postulating as many as 10 gene translocations and/or duplications and losses involving four protein-encoding genes (*nad3*,*nad4L*, *nad6*, and *nad1*) and six tRNAs genes (*trnN*, *trnS2*, *trnL*, *trnM*, *trnC* and *trnY*) [45]. Gene rearrangement in *S*. *maritima* is not as easily accommodated by the duplication, loss and translocation theory of gene rearrangement. To derive the *Strigamia maritima* mitochondrial gene order from the *Limulus polyphemus* ‘ground plan’ would require gene translocations involving five protein-coding genes (*cox3*, *cob*, *nad6*, *nad2* and *nad3*) and eleven tRNAs (*trnR*, *trnE*, *trnT*, *trnM*, *trnI*, *trnL2*, *trnY*, *trnV*, *trnS*, *trnN*, and *trnK*), and the observed order of these is not easily reached from the ancestral arrangement.

Alternative mechanisms for gene rearrangement may therefore be necessary in order to explain the gene order observed in *Strigamia*. As outlined, identifying tRNA sequences using computational analysis was made difficult by the asymmetry of their secondary stem and loop structure. A possible explanation for the novel gene order is that the mechanism for gene rearrangement in *Strigamia* relies on stem and loop structures [55, 56]. In vertebrates, the end-points of tandemly duplicated gene regions contain stem and loop structures: either from tRNAs or from the protein-coding gene regions [57]. More widely, in both vertebrates and invertebrates, tRNA genes are involved more frequently in mitochondrial gene rearrangements than protein-coding or ribosomal genes [27, 55]. It is possible that the asymmetrical and truncated structure of the *Strigamia* tRNAs leads to randomly located tandem repeats occurring simultaneously at many locations along the mitochondrial genome. Extensive gene rearrangement could alternatively be explained by small direct repeats. In ranid frogs, transpositions in terminal inverted or direct repeats have created non-functional copies of *trnL2* in the same position as the functional copy. Transposition of the repeated copy has subsequently resulted in a copy of trnL2 that is 5kb away from the ‘usual’ position observed in other vertebrates [56]. This pattern of rearrangement is similar to that observed in *Strigamia*, where *trnL2* is inverted from the ancestral position and has been translocated into the coding region, overlapping with *rrnS* and *rrnL*.

In our phylogenetic analysis, a sister-group relationship is weakly supported between Geophilomorpha (*Strigamia maritima*) and Scolopendromorpha (*Scolopocryptos sp*.) The mitochondrial gene order of *Scolopocryptos sp* has recently been shown as identical to that of the arthropod ‘ground plan’ represented by *Limulus polyphemus*, except for the interchanged positions of *trnL1* and *trnL2* [58] (Fig. 3). It appears, therefore, that no meaningful phylogenetic information can be derived from comparing the gene order of these two species, as any differences would be *Strigamia* specific. Further sequencing of mitochondrial genomes from additional members of the Geophilomorpha would show whether such extensive rearrangement is unique to *Strigamia* and hence a recent innovation or found commonly throughout this order of centipedes and hence a more ancient event. It is also apparent that the novel gene order found in the *Strigamia* mitochondrial genome contrasts with the exceptionally conservative gene content and arrangement observed in its nuclear genome [16].

### Phylogenetic Inference

Resolving the inter-relatedness of the Myriapoda, and determining their position within the Arthropoda, remains a difficult phylogenetic problem. In our Bayesian phylogeny (Fig. 4), the myriapods form a poorly resolved clade with the chelicerates, thus favouring the Myriochelata and Pancrustacea hypothesis over that of a monophyletic Mandibulata [19]. Our ML analysis (Fig. 5) resolves a paraphyletic Myriapoda, placing Chilopoda as sister group to Crustacea + Hexapoda, separate from the Diplopoda + Symphyla grouping. Pauropoda are resolved as sister group to the Chelicerata. SH-like branch support for the node splitting off (Diplopoda + Symphyla (Chilopoda (Crustacea + Hexapoda))) is only very low [0.22]. Phylogenies derived from mitochondrial DNA of other arthropod members have also resolved Myriochelata [20, 59, 60], but this is not always a well-supported grouping [20]. As mitochondrial DNA has a high A+T content—averaging approximately 70% in metazoan taxa—the likelihood of compositional bias and multiple substitutions means that phylogenies derived from mitochondrial genes are particularly prone to systematic error [24, 61]. Ecdysozoan phylogenies based on a much larger set of nuclear genes support a monophyletic Mandibulata and monophyletic Myriapoda once systematic error has been carefully dealt with [62]. It therefore seems probable that the data we have in this analysis is too small a sample, as well as possibly suffering from systematic error due to obvious compositional biases, to reconstruct these relationships accurately.

In this study, the centipedes, Chilopoda, are resolved as a monophyletic grouping, and the relationships within the order correspond with those derived from previous molecular analyses of nuclear ribosomal and nuclear protein-coding genes [33]. Scutigeromorpha, represented by *Scutigera coleoptrata* in our analysis, is found as the sister group to the three remaining centipede orders represented in our phylogeny (Lithobiomorpha, (*Bothropolys sp*., *Lithobius forficatus* and *Cermatobius longicornis*); Scolopendromorpha (*Scolopocryptos sp*.) and Geophilomorpha (*Strigamia maritima*)) with BPP = 1 and SH-like support = 0.99. Together with the Craterostigmomorpha—an order from which no mitochondrial genes have been sequenced—these three orders form the Pleurostigmomorpha. Our phylogenies therefore conform to the widely-held view that Scutigeromorpha are the ‘sister-order’ to the four remaining orders forming the Pleurostigmomorpha [33]. Our phylogenies also support the sister-group relationship between Geophilomorpha (*Strigamia maritima*) and Scolopendromorpha (*Scolopocryptos sp*.) with 0.77 BPP and 0.76 SH-like support in our ML phylogeny.

We sequenced the first complete mitochondrial genome of a geophilomorph centipede. Phylogenetic analyses using mitochondrial protein-coding genes were unable to support a monophyletic Mandibulata, but did support a monophyletic Chilopoda with inter-relatedness conforming to the view that Scutigeromorpha are the sister group to the four remaining chilopod orders comprising the Pleurostigmomorpha. Gene order of the *Strigamia* mitochondrial genome is unique compared to any other arthropod, or indeed any other metazoan, mitochondrial genome studied. This unusual organisation contrasts with the notably conservative nuclear genome [16]. Further sequencing and analysis of mitochondrial genomes from this order of centipedes is therefore required to see whether this unusual gene order is unique to *Strigamia*, or common to members of the Geophilomorpha.

## Supporting Information

S1 Table Primer pairs used for amplification of fragments within the mitochondrial genome of *Strigamia maritima*. (DOCX)

S2 Table GenBank accession numbers for taxa used in this study. (DOCX)
