# Supplementary material for: The Complete Mitochondrial Genome of the Geophilomorph Centipede Strigamia maritima
Source: PLoS One. 2015 Mar 20;10(3):e0121369. doi: 10.1371/journal.pone.0121369 (PMC4368715; doi:10.1371/journal.pone.0121369)
Supplement: S1 Table — (DOCX) [file pone.0121369.s001.docx]

| **PCR Fragment** | **Forward Primer** | **Forward Primer Sequence (5’-3’)** | **Reverse Primer** | **Reverse Primer Sequence (5’-3’)** |
| --- | --- | --- | --- | --- |
| ‘Closing’ the genome | Fw_17980 | AGCAGGTTCTGTCATTGGGTA | Rv_6744 | CCCCAGCCAAAAACTAACAA |
| I | Fw_407 | GTTAGTGTGGTTGGGGCTGT | Rv_2916 | AAAAATTCAAAATAACAAAAACACC |
| II | Fw_2001 | AGACCTCCGTATGCTTGGTG | Rv_4023 | ACACCACCCCCATAGTAGCA |
| III | Fw_3602 | TGAGGCTAGTGTGTGGGTCA | Rv_5543 | ATTCATTGCCCTCCCTTCTT |
| IV | Fw_5026 | GCTGCAGCAATTGGTCATAG | Rv_7287 | ACAGCCTAACCGACCAATCA |
| V | Fw_6632 | TCCTGTGGGGTCAAAGAATC | Rv_8460 | TGCTAAACAGGCGAGAACAA |
| VI | Fw_8151 | AGCCGCGTCTAGGGTTATCT | Rv_10457 | GCACGGCTACCATTTTCACT |
| VII | Fw_9701 | GCTGAGAGAAATCGCACTCC | Rv_12704 | AGGCACCCAAAGAAACAATG |
| IX | Fw_12448 | TTTTGTCGTATGGGGGTGAT | Rv_710 | CCGCCATATTACCAGCACTT |
| *nad6* | Fw_nad6 | CCCACCCTGTTGCTATTACC | Rv_nad6 | GCCCCTTATGTGCAGTTGTAA |

**Supporting Information**

**Table S1: Primer pairs used for amplification of fragments within the mitochondrial genome of *Strigamia maritima***
