# Supplementary material for: The Complete Mitochondrial Genome of the Geophilomorph Centipede Strigamia maritima
Source: PLoS One. 2015 Mar 20;10(3):e0121369. doi: 10.1371/journal.pone.0121369 (PMC4368715; doi:10.1371/journal.pone.0121369)
Supplement: S2 Table — (DOCX) [file pone.0121369.s002.docx]

**Supporting Information**

| **Classification** | **Species** | **Accession Number** |
| --- | --- | --- |
| **Myriapoda** |  |  |
| **Pauropoda** | *Pauropus longiramus* | HQ457012 |
| **Diplopoda** | *Antrokoreana gracilipes* | DQ344025 |
|  | *Narceus annularus* | AY055727 |
|  | *Thyropygus sp.* | AY055728 |
|  | *Abacion magnum* | JX437062 |
|  | *Appalachioria falcifera* | JX437063 |
|  | *Brachycybe lecontii* | JX437064 |
|  | *Sphaerotheriidae sp.* | JQ713564 |
| **Chilopoda** | *Scutigera coleoptrata* | AJ507061 |
|  | *Scolopocryptops sp.* | KC200076 |
|  | *Lithobius forficatus* | AF309492 |
|  | *Bothropolys sp.* | AY691655 |
|  | *Cermatobius longicornis* | KC155628 |
| **Symphyla** | *Symphylella sp.* | EF576853 |
|  | *Scutigerella causeyae* | DQ666065 |
| **Chelicerata** |  |  |
| **Arachnida** | *Heptathela hangzhouensis* | AY309258 |
|  | *Centruroides limpidus* | AY803353 |
| **Merostomata** | *Limulus polyphemus* | AF216203 |
| **Hexapoda** |  |  |
| **Insecta** | *Locusta migratoria* | X80245 |
|  | *Gryllotalpa orientalis* | AY660929 |
|  | *Lepidopsocidae sp.* | AF335994 |
|  | *Bombyx mori* | NC002355 |
|  | *Tribolium castaneum* | [AJ312413](http://www.ncbi.nlm.nih.gov/entrez/query.fcgi?cmd=search&db=nucleotide&doptcmdl=genbank&term=AJ312413%5Baccn%5D) |
|  | *Triatoma dimidiata* | AF301594 |
|  | *Tamolanica tamolana* | DQ241797 |
|  | *Periplaneta fuliginosa* | AB126004 |
|  | *Sclerophasma paresisense* | DQ241798 |
|  | *Drosophila melanogaster* | U37541 |
|  | *Pteronarcys princeps* | AY687866 |
|  | *Thermobia domestica* | AY639935 |
|  | *Tricholepidion gertschi* | AY191994 |
|  | *Petrobius brevistylis* | AY956355 |
|  | *Nesomachilis australica* | AY793551 |
| **Entognatha** | *Japyx solifugus* | AY771989 |
|  | *Gomphiocephalus hodgsoni* | AY191995 |
|  | *Podura aquatica* | AY639939 |
|  | *Tetrodontophora bielanensis* | AF272824 |
| **Crustacea** |  |  |
| **Malacostraca** | *Portunus trituberculatus* | AB093006 |
|  | *Penaeus monodon* | AF217843 |
|  | *Squilla empusa* | DQ191684 |
| **Branchiopoda** | *Artemia franciscana* | X69067 |
|  | *Triops cancriformis* | AB084514 |
|  | *Daphnia pulex* | AF117817 |
| **Maxillopoda** | *Megabalanus volcano* | AB167539 |
|  | *Tetraclita japonica* | AB126701 |
|  | *Pollicipes mitella* | AY514042 |
| **Priapula** | *Priapulus caudatus* | DQ463747 |
| **Mollusca** |  |  |
| **Scaphopoda** | *Siphonodentalium lobatum* | AY342055 |
| **Gasttopoda** | *Albinaria coerulea* | X83390 |
| **Brachiopoda** | *Laqueus rubellus* | AB035869 |
|  | *Terebratulina retusa* | AJ245743 |
| **Annelida** |  |  |
| **Polychaeta** | *Platynereis dumerilii* | AF178678 |
| **Chordata** |  |  |
| **Cephalochordata** | *Epigonichthys lucayanus* | AB110092 |

**Table S2: GenBank accession numbers for taxa used in this study**
